# Supplementary material for: Computational basis of hierarchical and counterfactual information processing
Source: Nat Hum Behav. 2025 Jun 11;9(9):1913–27. doi: 10.1038/s41562-025-02232-3 (PMC12454115; doi:10.1038/s41562-025-02232-3)
Supplement: Supplementary file 1 — Supplementary Figs. 1 and 2. [file 41562_2025_2232_MOESM1_ESM.pdf]

nature human behaviour

Supplementary information https://doi.org/10.1038/s41562-025-02232-3

Computational basis of hierarchical and counterfactual information processing

In the format provided by the authors and unedited

Supplementary Figures

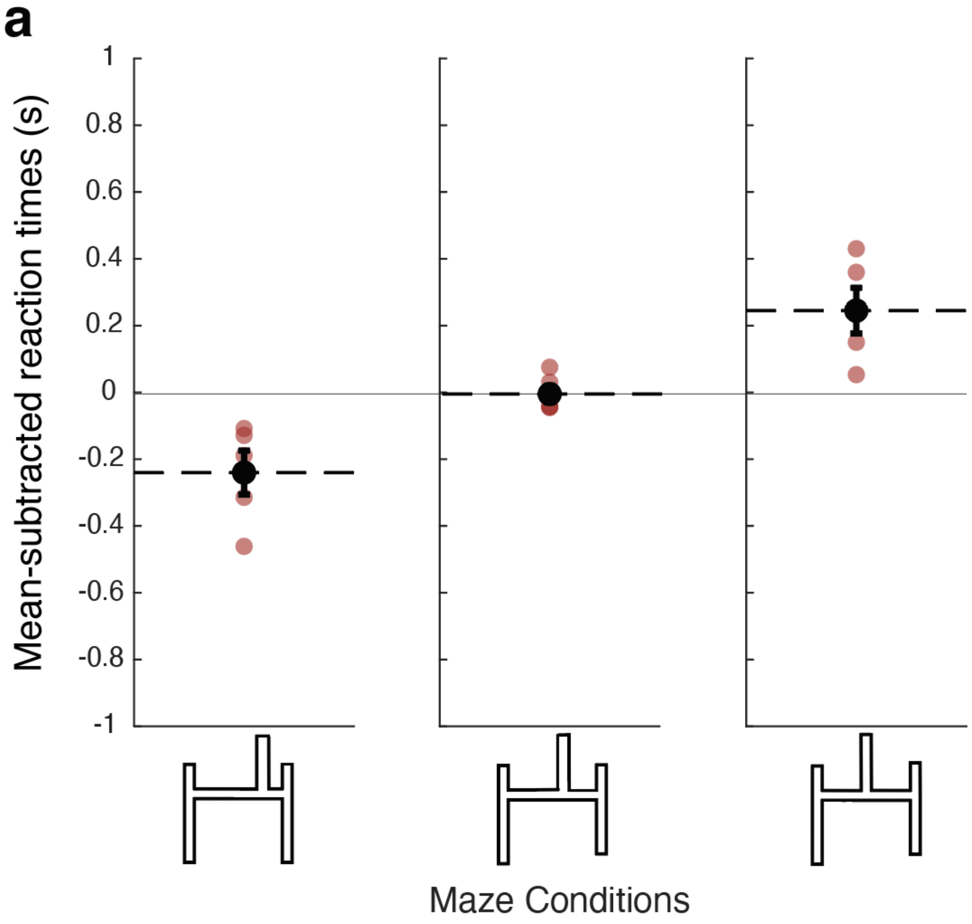

Supplementary Figure 1. Participants' reaction times on the Baseline H-maze. (a) Mean-subtracted reaction times (computed as the time from the third flash to the subject's choice) for individual human participants across the three horizontal arm difficulty maze conditions are shown as red circles. The average reaction time across participants for each maze condition is represented by a black circle, with the horizontal dashed line indicating the mean and black error bars showing the standard deviation (n=5).

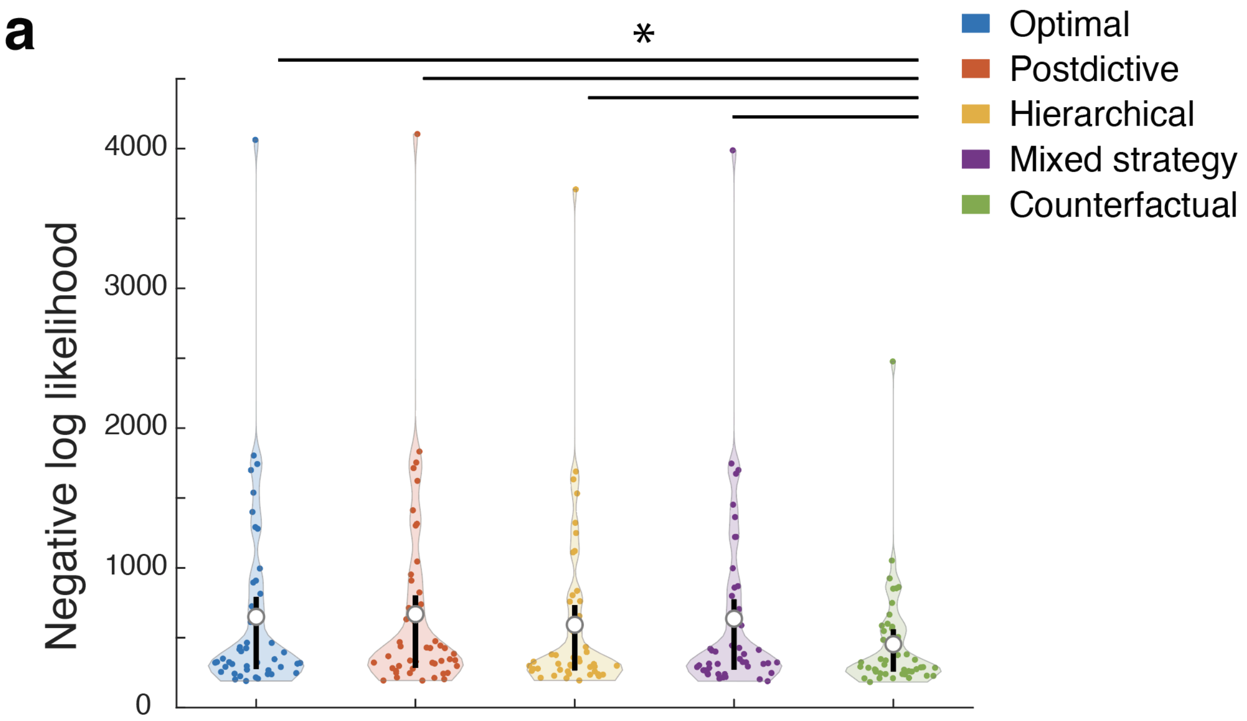

Supplementary Figure 2. Same as the main Figure 2d with the addition of a specific mixed strategy model (see Methods). The negative log likelihood of the counterfactual model was significantly lower than that of the other models (Left-tailed t-test, counterfactual vs. optimal: p=5.002e-5, counterfactual vs. postdictive: p=2.646e-5, counterfactual vs. hierarchical: p=2.924e-4, counterfactual vs. mixed: p=8.217e-5, n=47).
