# Supplementary material for: Computational basis of hierarchical and counterfactual information processing
Source: Nat Hum Behav. 2025 Jun 11;9(9):1913–27. doi: 10.1038/s41562-025-02232-3 (PMC12454115; doi:10.1038/s41562-025-02232-3)
Supplement: Supplementary file 2 — Reporting Summary [file 41562_2025_2232_MOESM2_ESM.pdf]

nature portfolio

Corresponding author(s): Mehrdad Jazayeri

Last updated by author(s): Sep 2, 2024

Reporting Summary

Nature Portfolio wishes to improve the reproducibility of the work that we publish. This form provides structure for consistency and transparency in reporting. For further information on Nature Portfolio policies, see our Editorial Policies and the Editorial Policy Checklist.

Statistics

For all statistical analyses, confirm that the following items are present in the figure legend, table legend, main text, or Methods section.

n/a	Confirmed	
☐	☒	The exact sample size (*n*) for each experimental group/condition, given as a discrete number and unit of measurement
☐	☒	A statement on whether measurements were taken from distinct samples or whether the same sample was measured repeatedly
☐	☒	The statistical test(s) used AND whether they are one- or two-sided<br>*Only common tests should be described solely by name; describe more complex techniques in the Methods section.*
☐	☒	A description of all covariates tested
☐	☒	A description of any assumptions or corrections, such as tests of normality and adjustment for multiple comparisons
☐	☒	A full description of the statistical parameters including central tendency (e.g. means) or other basic estimates (e.g. regression coefficient) AND variation (e.g. standard deviation) or associated estimates of uncertainty (e.g. confidence intervals)
☐	☒	For null hypothesis testing, the test statistic (e.g. *F*, *t*, *r*) with confidence intervals, effect sizes, degrees of freedom and *P* value noted<br>*Give P values as exact values whenever suitable.*
☐	☒	For Bayesian analysis, information on the choice of priors and Markov chain Monte Carlo settings
☐	☒	For hierarchical and complex designs, identification of the appropriate level for tests and full reporting of outcomes
☒	☐	Estimates of effect sizes (e.g. Cohen's *d*, Pearson's *r*), indicating how they were calculated

Our web collection on statistics for biologists contains articles on many of the points above.

Software and code

Policy information about availability of computer code

Data collection	Eyelink 1000, MWorks 0.8, Cognition.run, Prolific, jsPsych
Data analysis	Matlab R2022a, Python 3.9

For manuscripts utilizing custom algorithms or software that are central to the research but not yet described in published literature, software must be made available to editors and reviewers. We strongly encourage code deposition in a community repository (e.g. GitHub). See the Nature Portfolio guidelines for submitting code & software for further information.

Data

Policy information about availability of data

All manuscripts must include a data availability statement. This statement should provide the following information, where applicable:

- Accession codes, unique identifiers, or web links for publicly available datasets
- A description of any restrictions on data availability
- For clinical datasets or third party data, please ensure that the statement adheres to our policy

https://github.com/jazlab/MR_CT_NW_MJ_2024/tree/master

Research involving human participants, their data, or biological material

Policy information about studies with human participants or human data. See also policy information about sex, gender (identity/presentation), and sexual orientation and race, ethnicity and racism.

Reporting on sex and gender	93 males and 68 females. The sex is randomly sampled in the pool of participants available on Prolific. We have no hypothesis on sex-based difference in the task and have no such analysis in the study.
Reporting on race, ethnicity, or other socially relevant groupings	We have no socially relevant categorization variables in the study.
Population characteristics	See above
Recruitment	We have no filter on participants other than 'fluent English reading'. We published 150 openings of experiment opportunities on Prolific, and qualified participants on Prolific voluntarily participated.<br>The experiments do not require specific skills or backgrounds, and we don't expect any self-selection biases or others.
Ethics oversight	Committee on the Use of Humans as Experimental Subjects at the Massachusetts Institute of Technology

Note that full information on the approval of the study protocol must also be provided in the manuscript.

Field-specific reporting

Please select the one below that is the best fit for your research. If you are not sure, read the appropriate sections before making your selection.

☐ Life sciences ☒ Behavioural & social sciences ☐ Ecological, evolutionary & environmental sciences

For a reference copy of the document with all sections, see nature.com/documents/nr-reporting-summary-flat.pdf

Behavioural & social sciences study design

All studies must disclose on these points even when the disclosure is negative.

Study description	mixed-methods case study on human decision-making strategy for hierarchical problems
Research sample	Randomly sampled participants on Prolific. 93 males and 68 females, 18-70 years old. We have no hypothesis on the demographic influence in this study.<br>The sample size of the pre-registered online experiment was chosen empirically to be 10 times the on-site experiment sample size for reliable reproduction. The samples are representative without identifiable biases.
Sampling strategy	Random sample. We first peformed the experiment with n=15 on-site participants. Then pre-registered and verified the results on n=150 online participants.<br>We didn't calculate the sample size through statistical methods. The sample size of the pre-registered online experiment was chosen empirically to be 10 times the on-site experiment sample size for reliable reproduction.
Data collection	For on-site experiment, stimuli and behavioral contingencies were controlled by an open-source software (MWorks; mworks-project.org/) running on an Apple Macintosh platform. Eye tracking data were collected by Eyelink device. No researcher was present besides the participants and the researcher was blind to the experiment during data collection.<br>For online experiment, the tasks were coded in jsPsych (www.jspsych.org) and deployed on the Cognition.run (www.cognition.run) platform. We only collected the keyboard response from online participants.
Timing	On-site experiment: HMaze data was collected 10/20/2018 to 11/02/2018. The variants were collected 9/16/2019 to 10/18/2019.<br>Online experiment: created and finished on 28 Feb 2024
Data exclusions	To ensure the quality of online participant data, we exclude subjects whose measured Weber fraction w on the control T-maze experiment or condition 1 of experiment 2 exceeds 0.4.
Non-participation	17 participant returned their response due to technical problems, 1 participant was dropped out due to time-out
Randomization	not allocated into groups

Reporting for specific materials, systems and methods

We require information from authors about some types of materials, experimental systems and methods used in many studies. Here, indicate whether each material, system or method listed is relevant to your study. If you are not sure if a list item applies to your research, read the appropriate section before selecting a response.

Materials & experimental systems

n/a	Involved in the study
☒	☐ Antibodies
☒	☐ Eukaryotic cell lines
☒	☐ Palaeontology and archaeology
☒	☐ Animals and other organisms
☒	☐ Clinical data
☒	☐ Dual use research of concern
☒	☐ Plants

Methods

n/a	Involved in the study
☒	☐ ChIP-seq
☒	☐ Flow cytometry
☒	☐ MRI-based neuroimaging

Plants

Seed stocks	*Report on the source of all seed stocks or other plant material used. If applicable, state the seed stock centre and catalogue number. If plant specimens were collected from the field, describe the collection location, date and sampling procedures.*
Novel plant genotypes	*Describe the methods by which all novel plant genotypes were produced. This includes those generated by transgenic approaches, gene editing, chemical/radiation-based mutagenesis and hybridization. For transgenic lines, describe the transformation method, the number of independent lines analyzed and the generation upon which experiments were performed. For gene-edited lines, describe the editor used, the endogenous sequence targeted for editing, the targeting guide RNA sequence (if applicable) and how the editor was applied.*
Authentication	*Describe any authentication procedures for each seed stock used or novel genotype generated. Describe any experiments used to assess the effect of a mutation and, where applicable, how potential secondary effects (e.g. second site T-DNA insertions, mosiacism, off-target gene editing) were examined.*
